# Leptin, but not ghrelin, is associated with food addiction scores in a population-based subject sample

**DOI:** 10.3389/fpsyt.2023.1200021

**Published:** 2023-07-25

**Authors:** Dirk Alexander Wittekind, Jürgen Kratzsch, Roland Mergl, Ronny Baber, Kerstin Wirkner, Matthias L. Schroeter, A. Veronica Witte, Arno Villringer, Michael Kluge

**Affiliations:** ^1^Institute of Laboratory Medicine, Clinical Chemistry and Molecular Diagnostics, University of Leipzig, Leipzig, Germany; ^2^Institute of Psychology, University of the Bundeswehr Munich, Neubiberg, Germany; ^3^Leipzig Research Center for Civilization Diseases (LIFE), University of Leipzig, Leipzig, Germany; ^4^Department of Neurology, Clinic of Cognitive Neurology, Max Planck Institute for Cognitive and Brain Sciences, University of Leipzig, Leipzig, Germany; ^5^Department of Psychiatry and Psychotherapy, University of Leipzig, Leipzig, Germany; ^6^Department of Psychiatry, Rudolf-Virchow-Klinikum Glauchau, Glauchau, Germany

**Keywords:** ghrelin, leptin, Yale Food Addiction scale, food addiction, obesity

## Abstract

**Background:**

Ghrelin and leptin are both peptide hormones and act as opposing players in the regulation of hunger, satiety and energy expenditure. Leptin reduces appetite and feelings of hunger and is secreted mainly by adipocytes, while ghrelin increases appetite and food intake and reduces metabolic rate. Both hormones have been implicated in addictive disorders. Ghrelin was shown to have pro-addictive effects while leptin’s role in addiction yields more conflicting results. Their involvement in the regulation of both food intake and addictive behaviors make them interesting candidates when investigating the regulation of food addiction. However, only few human studies have been performed and large-scale studies are lacking to date. We aimed to investigate the association between total ghrelin and leptin serum levels with scores in the Yale Food Addiction Scale (YFAS).

**Methods:**

Subjects were recruited in the LIFE Adult cohort. 909 subjects were included in the analysis and we performed univariate multiple linear regression models, adjusted for age, sex (in total group analyses only), alcohol consumption, smoking status, BMI scores, cortisol concentrations, Center for Epidemiological Studies Depression Scale (CES-D) and the 7-item Generalized Anxiety Disorder Scale (GAD-7) sum scores. The dependent variable was the YFAS score.

**Results:**

In men, leptin serum levels showed a significant positive association (standardized *β* = 0.146; *p* = 0.012) with the YFAS score. This finding was confirmed in an extreme-group comparison: men in the highest quartile of leptin levels had significantly higher YFAS sum scores than men in the lowest quartile (1.55 vs. 1.18; *p* = 0.00014). There was no association with YFAS sum score in the total group (standardized *β* = −0.002; *p* = 0.974) or in women (standardized *β* = −0.034; *p* = 0.674). Total serum ghrelin showed no association with YFAS sum score neither in the total group (standardized *β* = −0.043; *p* = 0.196) nor in men (*n* = 530; standardized *β* = −0.063; *p* = 0.135) or women (*n* = 379; standardized *β* = −0.035; *p* = 0.494).

**Conclusion:**

Our findings are in line with previous literature and suggest that total ghrelin serum levels are not associated with food addiction scores. Leptin had been previously shown to be associated with food addiction and we confirmed this finding for men in a large, population-based approach.

## 1. Introduction

Obesity is a global health risk with increasing numbers of overweight and obese individuals (1). Both for Western and developing countries, studies forecast that this problem will further increase in the near future, leading to increasing healthcare, societal and individual costs (1). The underlying pathophysiological mechanisms are highly complex (2) and are only partially understood thus far. In recent years, the concept of Food Addiction (FA) has gained considerable attention and an increasing number of studies suggest that addiction mechanisms might contribute to growing rates of obesity (3). A relevant percentage of about 24% of overweight and obese individuals show signs of Food Addiction (FA) (3). FA is a relatively new concept that suggests that specific eating behaviors can show patterns of addictive behavior, like excessive consumption, loss of control and craving (3, 4). It is based on the observation that the consumption of highly palatable foods, i.e., high in fat- sugar- and salt content, can have similar effects on the brain as addictive substances, activating reward circuits that can lead to addictive behaviors in some individuals (5). Like gambling, FA is considered a behavioral addiction by many authors (4), while others categorize it as a substance addiction (6). It has not been recognized as a distinct diagnosis in the Diagnostic and Statistical Manual of Mental Disorders, Fifth Edition [DSM-5; (7)] or the International Classification of Diseases and Related Health Problems, 10th Edition [ICD-10; (8)]. As such, it is comparable to conditions like shopping addiction or internet addiction (9). While having a high comorbidity with Binge-Eating-Disorder (BED), FA and BED are most likely two distinct, yet overlapping, entities (4, 10).

Orexigenic 28-amino-acid peptide hormone ghrelin, mostly synthesized in times of stress and hunger in the stomach (11–13) and the adipokine leptin are involved in the brain’s reward circuits (14–16). Ghrelin promotes food intake especially of highly palatable foods via the interaction with the brain’s reward system (14, 16, 17) and increasing evidence strongly suggests that it is involved in the pathogenesis of substance-related addictive disorders, especially alcohol dependence (14, 17–19). Interestingly, food-related secretion patterns and effects of ghrelin on food-intake in healthy subjects are very similar to its alcohol-related secretion patterns: Ghrelin levels are high during fasting and increase when anticipating food intake, e.g., before lunch (13) or when being presented a food cue (20) and decrease after food intake. Likewise, ghrelin levels increase when anticipating ingestion of alcohol and decrease after alcohol consumption (14, 21, 22). For leptin, a peptide hormone produced in adipocytes (23), evidence is scarcer and a lot less conclusive, but leptin receptors are expressed in the mesolimbic dopaminergic reward system, including the ventral tegmental area (VTA) and the nucleus accumbens (NAc) (16, 24). In humans, studies show leptin to be associated with craving in nicotine and cocaine dependence (25–27) and leptin gene polymorphisms were associated with measures in alcohol use (28).

Thus, investigating the association of ghrelin and leptin levels with FA scores might allow conclusion as to whether these peptides are involved in FA development. To date, only few studies have investigated the association between FA and ghrelin. Associations were studied in obese patient samples (29, 30), patients with Major Depressive Disorder [MDD; (31)] and healthy female subjects (32). Results found no significant association in three studies (29, 31, 32) and one study described a positive association between FA scores and ghrelin serum levels (30). To date, studies with large sample sizes are lacking and no study with a population-based approach has been published to our knowledge.

Altogether seven studies assessed the association between leptin and FA with mixed results (33). While three studies found a positive relationship (31, 34, 35), four studies did not find a significant association (5, 29, 31, 32). However, study samples were heterogeneous: patients with MDD (5, 31), adolescent psychiatric inpatients (35), and obese patients as well as healthy controls were investigated (29, 32, 34).

To address the need for further large association studies, we aimed to investigate the association between total serum ghrelin levels and leptin levels with FA-scores in a large, population based approach, using the established Yale Food Addiction Scale (YFAS) to quantify FA scores.

First, a confirmatory testing approach was selected for the investigation of the primary hypotheses that YFAS sum scores were significantly associated with ghrelin and leptin serum concentrations if the effects of several covariables were controlled for. Second, the examination of sex differences was independently done within an exploratory approach, as evidence for sex-specific effects exists for both ghrelin (36) and leptin (37).

## 2. Methods

### 2.1. Study design and subjects

Data for the present analysis come from the LIFE-Adult-Study (Leipzig Research Center for Civilization Diseases), a large population-based cohort study comprising 10,000 adults (mainly within the age range 40–79 years), who were randomly recruited in Leipzig, a large city in Germany (38). All participants gave written informed consent to participate in the study. The study was conducted according to the Declaration of Helsinki and approved by the ethics committee of the University of Leipzig (registration-number: 263–2009-14,122,009). Blood samples were collected after an overnight fast (at least 8 h of fasting) including abstinence from smoking, between 07:30 and 10:30 h. The blood samples had been immediately processed by the team of the LIFE pre-analytical laboratory which is part of the Leipzig Medical Biobank and sent directly to the Institute of Laboratory Medicine, Clinical Chemistry, and Molecular Diagnostics (ILM) where direct analyses were carried out. Additional samples were frozen within 2 h after blood withdrawal in the vapor phase of liquid nitrogen at temperatures below – 150°C in Askion c-line HS200S (Askion, Gera, Germany) until usage for ghrelin measurements.

The selection of subjects for the ghrelin analysis were based on availability of serum samples, overlap with MRI-scans and a representation of different age groups (18–40 years, 40–60 years and above 60 years). Beyond that, subjects were selected by chance. Inclusion criteria were: Valid measurements of total ghrelin; complete data regarding age, sex, alcohol consumption, nicotine consumption, BMI score, cortisol concentration, GAD-7 and CES-D sum scores; written informed consent. Exclusion criteria (due to possible effects on ghrelin levels) were as follows: current treatment due to an autoimmune disease, treatment in the last year because of a diagnosis of cancer, current diseases of the gastrointestinal system, a history of stroke, multiple sclerosis or epilepsy. Valid measurements of total ghrelin were available for 1,666 LIFE participants. 757 subjects were included for not meeting in−/ exclusion criteria (712 of which had incomplete data sets). Thus, the final sample consisted of 909 persons (530 men and 379 women).

### 2.2. Ghrelin measurements

All ghrelin measurements were done in serum by the use of a radioimmunoassay for total ghrelin (Mediagnost, Reutlingen Germany). Samples were not pre-treated with enzyme inhibitors or acidification. Due to this, only total ghrelin was measured, as it is much more stable than acyl-ghrelin. Sensitivity of the assay was 0.04 ng/mL, mean intra-assay coefficients of variation were 2.7–4.3%; interassay coefficients of variation were between 6.9 and 9.2% for the mean expected range of clinical data around 0.88 and 0.97 ng/mL.

### 2.3. Leptin measurements

Serum leptin concentrations were measured with a sandwich ELISA by Mediagnost GmbH (E07, Reutlingen, Germany) with intra-assay coefficients of variation between 4.7 and 5.2%, inter-assay coefficients of variation between 4.4 and 4.8% and a limit of detection of 0.25 μg/L.

### 2.4. Cortisol measurements

Cortisol measurements were performed by the cobas^®^ e601 fully automated system (Roche Diagnostics, Penzberg, Germany). Interassay coefficients of variation of 4.03 and 2.80% with cortisol levels between 65.6–69.2 nmol/L (*n* = 174) and 762–807 nmol/L (*n* = 174) were calculated after representative measurement of quality control sera over 6 months.

### 2.5. Yale Food Addiction Scale

Food addiction symptoms were assessed using the first edition of the Yale Food Addiction scale (YFAS) (39). The newer version YFAS 2.0 was not yet available when the study was conducted. This questionnaire applies the DSM-IV criteria for substance dependence to eating behavior. We selected the continuous food addiction symptom score ranging from 0 to 7 as the primary variable of interest in our analyses due to the low prevalence of manifest FA in our sample (see below). Food addiction, like other psychopathological traits, might be better represented in a continuum (40, 41).

### 2.6. Acquisition of data on tobacco and alcohol consumption

Tobacco consumption was assessed via self-administered questionnaire and interview. Subjects were grouped in three categories: active smoker, former smoker and never-smoker. Active smokers were considered all those participants who had smoked regularly for at least 6 months consecutively in their lifetime and at least occasionally at the time of examination. Subjects who had smoked continuously for more than 6 months during their lifetime, but were not smoking at the time of assessment, were defined as former smokers (42).

Frequency and amount of consumption of alcoholic beverages (i.e., beer/wine/spirits) during the last 12 months were semiquantitatively assessed using a self-administered food frequency and alcohol questionnaire (FFQ). Possible answers for the frequency of alcohol consumption were “multiple times a day,” “daily,” “multiple times a week,” “once a week,” “two to three times a month,” “once a month or rarer” or “almost never.” Also, the amount of beverage consumption was assessed by defined categories. From the amount and frequency of alcoholic beverage as well as the average alcohol content of different beverages, the average consumption of pure alcohol (g/day) was calculated (18).

### 2.7. Assessment of BMI

The BMI is defined as the body weight divided by the square of the body height (kg/m^2^). Body weight was measured with an electronic scale (SECA 701, Seca GmbH & Co KG) with a precision of 0.01 kg, height using a stadiometer (SECA 240) to the nearest 0.1 cm by trained staff according to standardized protocols.

### 2.8. Statistical analysis

For confirmatory testing of the primary hypotheses, univariate multiple linear regression analyses were computed for the total sample, with the dependent variable being the YFAS sum score. In the first set of regression analyses, the independent variable was the ghrelin serum concentration; in the second set, it was the leptin serum concentration. The analyses were adjusted for the following variables (as these variables have been shown to be associated with ghrelin levels): sex (12, 43), age (12, 44, 45), alcohol consumption (18), smoking status (42), BMI (12), cortisol levels (12, 46), depression (47) and GAD-7 sum scores (48).

Regression coefficients (*β*) including 95% confidence intervals (CI) were calculated in this context.

In a secondary exploratory approach, corresponding subgroup analyses in men and women were conducted; in this context, the same covariables were selected (without sex). Analogous analyses were performed for the YFAS sub-category scores regarding “amount” (sum of the YFAS items 1, 2, and 3 – Substance taken in larger amount and for longer period than intended), “desire” (sum of the YFAS items 4, 22, 24, and 25 – Persistent desire or repeated unsuccessful attempts to quit) and “continuous use” (YFAS item 19 – Use continues despite knowledge of adverse consequences (e.g., failure to fulfill role obligation, use when physically hazardous)).

In addition, extreme-group comparisons were performed: We tested for statistical differences of YFAS sum scores between the highest and lowest quartile according to ghrelin and leptin serum levels, separated for men and women, by using a Mann–Whitney-U-test.

Food addiction was defined as the presence of clinically significant impairments regarding food intake combined with a YFAS sum score of at least 3. The percentage of food addiction was calculated for the total sample as well as men and women separately. By using Fisher’s exact test, it was tested whether the percentages of food addiction in men and women were statistically significant. Moreover, mean ghrelin and leptin concentrations in individuals with versus without food addiction were computed. In view of the small corresponding numbers (see below) only descriptive statistics were presented in this context.

The SPSS software (version 26.0) was chosen for the statistical analyses. The significance level was set at α = 0.05. In case of multiple testing the Bonferroni correction has been applied as follows: The correction was made for ghrelin and leptin separately resulting in three tests (for the whole sample; males and females, respectively). Thus, the adjusted threshold for significance was 0.0167 (=0.05/3). All statistical tests were two-tailed.

## 3. Results

### 3.1. Characteristics of the final sample

Demographic characteristics (like age and sex distribution) as well as clinical characteristics of the final sample (especially severity of food addiction, depression and anxiety) are presented in Table 1.

**Table 1:** Description of the sample.

	Total sample (*n* = 909)	Males (*n* = 530)	Females (*n* = 379)	Value of *p*
Total YFAS sum score, mean (SD)	1.34 (0.84)	1.30 (0.75)	1.40 (0.94)	0.311^a^
YFAS sum score for “desire,” mean (SD)	1.38 (0.71)	1.36 (0.68)	1.41 (0.76)	0.423^a^
YFAS sum score for “amount,” mean (SD)	0.05 (0.29)	0.03 (0.21)	0.08 (0.37)	0.015*^a^
YFAS sum score for “continuous use,” mean (SD)	0.20 (0.40)	0.19 (0.39)	0.22 (0.41)	0.304^a^
Ghrelin serum concentration, pg./ml, mean (SD)	900.76 (436.52)	810.53 (317.97)	1026.94 (537.45)	6.30*10^−15^***^a^
Leptin serum concentration, μg/l, mean (SD)	11.33 (11.82)	6.36 (6.07)	18.29 (14.17)	4.93*10^−70^***^a^
Age, years, mean (SD)	56.77 (16.47)	57.40 (16.62)	55.90 (16.23)	0.038*^a^
BMI, kg/m^2^, mean (SD)	27.02 (4.47)	27.33 (4.01)	26.59 (5.01)	0.00027***^a^
Non-smokers, %	787 (86.58%)	445 (83.96%)	342 (90.24%)	0.006**^b^
Active smokers, %	122 (13.42%)	85 (16.04%)	37 (9.76%)	---
Alcohol consumption, g/day, mean (SD)	12.62 (17.50)	17.60 (19.94)	5.64 (9.79)	9.02*10^−35^***^a^
Cortisol concentration, nmol/l, mean (SD)	515.12 (198.80)	501.48 (145.10)	534.19 (254.64)	**0.357**^**a**^
CES-D sum score, mean (SD)	9.25 (6.08)	8.59 (5.19)	10.18 (7.06)	0.002**^a^
GAD-7 sum score, mean (SD)	2.70 (2.74)	2.44 (2.60)	3.07 (2.88)	0.00084***^a^

Amount: sum of the YFAS items 1, 2, and 3 – Substance taken in larger amount and for longer period than intended; BMI: Body Mass Index; CES-D, Center for Epidemiological Studies Depression Scale (49, 50). CI, confidence interval; continuous use: YFAS item 19 – Use continues despite knowledge of adverse consequences (e.g., failure to fulfill role obligation, use when physically hazardous); desire: sum of the YFAS items 4, 22, 24, and 25 – Persistent desire or repeated unsuccessful attempts to quit; GAD-7, Generalized Anxiety Disorder 7-item Scale (51, 52); SD, Standard Deviation; YFAS, Yale Food Addiction Scale (39). *p ≤ 0.05; **p ≤ 0.01; ***p ≤ 0.001. Significant findings were marked in bold. ^a^Due to non-normal distribution of the dependent variables (Kolmogorov–Smirnov-Test: *p* < 0.05), a Mann–Whitney U test was computed in order to calculate *p* values for sex differences regarding total YFAS sum scores (*Z* = −1.01), YFAS sum scores for desire (*Z* = −0.80), amount (*Z* = −2.44) and continuous use (*Z* = −1.03), ghrelin serum concentrations (*Z* = −7.80), leptin serum concentrations (*Z* = −17.69), age (*Z* = −2.08), BMI scores (*Z* = −3.64), alcohol consumption (*Z* = −12.30), cortisol concentration (*Z* = −0.92), CES-D sum scores (*Z* = −3.04) and GAD-7 sum scores (*Z* = −3.34). ^b^A chi^2^ test for a two-by-two cross table (*χ*^2^ = 7.49; df = 1) was applied.

10 of 903 individuals (1.11%; *n* = 903 due to 6 cases without information about clinically significant impairments associated with food intake) fulfilled the criteria of food addiction. Food addiction was present in 5 of 526 men (0.95%) and 5 of 377 women (1.33%); the corresponding sex difference failed to be statistically significant (*p* = 0.749).

### 3.2. Association between ghrelin serum levels and the intensity of food addiction

Total serum ghrelin levels showed no statistically significant association with the intensity of food addiction as measured by the YFAS sum score – neither in the total group (standardized *β* = −0.043; *p* = 0.196) nor in men (*n* = 530; standardized *β* = −0.063; *p* = 0.135) or women (*n* = 379; standardized *β* = −0.035; *p* = 0.494) (for details see Table 2).

**Table 2:** Results of univariate multiple linear regression analyses regarding the association between ghrelin serum levels and YFAS sum scores reflecting the intensity of food addiction in participants of the study.

Variables	Regression coefficient *β* (95% CI)	Standardized *β*	*t*	*p* value
YFAS sum scores in the total sample (*N* = 909) Corrected *R*^2^ = 0.089; *F* = 10.88; *p* < 0.001***				
Intercept	0.431 (−0.063; 0.925)	---	1.713	0.087^+^
Ghrelin serum levels	−0.00008 (−0.00021; 0.00004)	−0.043	−1.293	0.196
Age	−0.008 (−0.012; −0.005)	−0.163	−4.670	3.5*10^−6^***
Sex	0.118 (0.0002; 0.237)	0.070	1.966	0.0496*
Alcohol consumption	0.002 (−0.001; 0.005)	0.046	1.333	0.183
Smoking Status	0.035 (−0.041; 0.110)	0.030	0.899	0.369
BMI	0.036 (0.023; 0.049)	0.193	5.508	4.7*10^−8^***
Cortisol concentration	−0.00004 (−0.0003; 0.0002)	−0.009	−0.250	0.803
CES-D sum score	0.019 (0.009; 0.030)	0.141	3.661	0.00027***
GAD-7 sum score	0.027 (0.003; 0.050)	0.087	2.257	**0.024** *
Men (*n* = 530) Corrected *R*^2^ = 0.109; *F* = 9.08; *p* < 0.001***				
Intercept	0.355 (−0.245; 0.956)	---	1.163	0.245
Ghrelin serum levels	−0.00015 (−0.00035; 0.00005)	−0.063	−1.496	0.135
Age	−0.008 (−0.012; −0.005)	−0.185	−4.250	2.5*10^−5^***
Alcohol consumption	0.00008 (−0.003; 0.003)	0.002	0.047	0.962
Smoking Status	0.045 (−0.040; 0.129)	0.043	1.030	0.303
BMI	0.048 (0.031; 0.065)	0.256	5.614	3.2*10^−8^***
Cortisol concentration	−0.0001 (−0.0006; 0.0003)	−0.023	−0.539	0.590
CES-D sum score	0.018 (0.004; 0.033)	0.127	2.582	**0.010** **
GAD-7 sum score	0.027 (−0.001; 0.055)	0.092	1.874	0.061^+^
Women (*n* = 379) Corrected *R*^2^ = 0.074; *F* = 4.78; *p* < 0.001***				
Intercept	0.875 (0.062; 1.688)	---	2.117	0.035*
Ghrelin serum levels	−0.00006 (−0.00024; 0.00011)	−0.035	−0.684	0.494
Age	−0.008 (−0.015; −0.001)	−0.141	−2.381	**0.018** *
Alcohol consumption	0.013 (0.003; 0.023)	0.136	2.662	**0.008** **
Smoking Status	0.004 (−0.139; 0.148)	0.003	0.059	0.953
BMI	0.027 (0.007; 0.048)	0.146	2.660	**0.008** **
Cortisol concentration	−0.00009 (−0.0005; 0.0003)	−0.025	−0.431	0.667
CES-D sum score	0.018 (0.002; 0.034)	0.136	2.248	**0.025** *
GAD-7 sum score	0.033 (−0.006; 0.073)	0.103	1.680	**0.094**

b, Regression coefficient; *β*, Standardized regression coefficient; BMI, Body Mass Index; CES-D, Center for Epidemiological Studies Depression Scale (49, 50); CI, confidence interval; GAD-7, Generalized Anxiety Disorder 7-item Scale (51, 52); N/n, sample sizes; YFAS, Yale Food Addiction Scale (39). *p ≤ 0.05; **p ≤ 0.01; ***p ≤ 0.001. Significant findings were marked in bold.

Regarding the YFAS subscale sum scores for desire, amount and continuous use total serum ghrelin concentrations were not significantly associated with them in the total sample. The same was true for the subgroups of men and women.

All univariate multiple linear regression models have been adjusted for age, sex (only in the total sample), alcohol consumption, smoking status (1 = active smoking; 0 = non-smoking), BMI scores, cortisol concentrations, CES-D sum scores, and GAD-7 sum scores.

These findings were in line with the results of extreme group comparisons: Neither in men nor in women individuals with the highest quartile of ghrelin serum concentrations had YFAS sum scores which were significantly different from those in individuals with the lowest quartile of ghrelin serum levels (see Table 3).

**Table 3:** Extreme-group-comparison of food addiction intensity in the highest and lowest quartiles of ghrelin serum levels.

Quartiles	Ghrelin serum levels in pg./mL		Mean YFAS scores (SD) (range)		*N*	
	Men	Women	Men	Women	Men	Women
Lowest: <	612.00	689.00	1.31 (0.73) (0–4)	1.43 (0.95) (0–6)	133	96
Highest: >	917.50	1,252.00	1.19 (0.72) (0–6)	1.29 (0.84) (0–5)	132	95
---	---	---	*Z* = −1.493 ^a^*p* = 0.135	*Z* = −0.781 ^a^*p* = 0.435	---	---

SD, Standard Deviation; YFAS, Yale Food Addiction Scale (39). ^a^A Mann–Whitney U test was performed to assess group differences in YFAS scores due to non-normal distribution of the dependent variable (Kolmogorov–Smirnov-Test: *p* < 0.05).

Ten individuals met the diagnostic criteria for food addiction (male = 5; female = 5). Individuals with FA were found to have higher ghrelin serum concentrations (mean = 1026.60 pg./mL; SD = 542.73 pg./mL) than individuals without food addiction (mean = 900.06 pg./mL; SD = 435.88 pg./mL; *n* = 893). In contrast to this finding, men with food addiction (*n* = 5) had lower ghrelin serum levels (mean = 703.60 pg./mL; SD = 97.87 pg./mL) than men without food addiction (mean = 811.35 pg./mL; SD = 318.64 pg./mL; *n* = 521) whereas women with food addiction (*n* = 5) had higher ghrelin serum levels (mean = 1349.60 pg./mL; SD = 626.38 pg./mL) than women without food addiction (mean = 1024.31 pg./mL; SD = 536.62 pg./mL; *n* = 372).

### 3.3. Association between leptin serum levels and the intensity of food addiction

In the total group, there was no statistically significant association between the corresponding serum levels and the YFAS sum score (standardized *β* = 0.002; *p* = 0.974) or women (standardized *β* = −0.034; *p* = 0.674). In men, there was a significantly positive association between these variables (standardized *β* = 0.146; *p* = 0.012) (see Table 4), which was also significant after Bonferroni correction for multiple testing.

**Table 4:** Results of multiple linear regression analyses regarding the association between leptin serum levels and YFAS sum scores reflecting the intensity of food addiction in participants of the study.

Variables	Regression coefficient *β* (95% CI)	Standardized *β*	*t*	*p* value
YFAS sum scores in the total sample (*N* = 909) Corrected *R*^2^ = 0.087; *F* = 10.67; *p* < 0.001***				
Intercept	0.345 (−0.263; 0.954)	---	1.114	0.266
Leptin serum levels	0.0001 (−0.007; 0.008)	0.002	0.032	0.974
Age	−0.008 (−0.012; −0.005)	−0.164	−4.651	3.8*10^−6^***
Sex	0.098 (−0.052; 0.247)	0.058	1.282	0.200
Alcohol consumption	0.002 (−0.001; 0.005)	0.043	1.255	0.210
Smoking Status	0.032 (−0.044; 0.107)	0.027	0.823	0.411
BMI	0.038 (0.020; 0.056)	0.201	4.085	4.8*10^−5^***
Cortisol concentration	−0.00002 (−0.0003; 0.0003)	−0.004	−0.123	0.902
CES-D sum score	0.019 (0.009; 0.030)	0.141	3.672	**0.00025** ***
GAD-7 sum score	0.026 (0.002; 0.049)	0.083	2.171	**0.030** *
Men (*n* = 530) Corrected *R*^2^ = 0.116; *F* = 9.66; *p* < 0.001***				
Intercept	0.592 (−0.047; 1.231)	---	1.821	0.069
Leptin serum levels	0.0181 (0.004; 0.032)	0.146	2.507	**0.012** *
Age	−0.008 (−0.012; −0.004)	−0.182	−4.196	3.2*10^−5^***
Alcohol consumption	−0.0002 (−0.003; 0.003)	−0.006	−0.140	0.889
Smoking Status	0.035 (−0.050; 0.119)	0.034	0.807	0.420
BMI	0.031 (0.008; 0.053)	0.164	2.688	**0.007** **
Cortisol concentration	−0.00009 (−0.0005; 0.0003)	−0.017	−0.405	0.685
CES-D sum score	0.018 (0.004; 0.032)	0.125	2.548	**0.011** *
GAD-7 sum score	0.026 (−0.002; 0.054)	0.090	1.842	0.066^+^
Women (*n* = 379) Corrected *R*^2^ = 0.073; *F* = 4.74; *p* < 0.001***				
Intercept	0.686 (−0.173; 1.545)	---	1.571	0.117
Leptin serum levels	−0.0022 (−0.013; 0,008)	−0.034	−0.421	0.674
Age	−0.008 (−0.015; −0.002)	−0.146	−2.427	**0.016** *
Alcohol consumption	0.013 (0.003; 0.023)	0.135	2.647	**0.008** **
Smoking Status	0.002 (−0.142; 0.146)	0.001	0.027	0.978
BMI	0.034 (0.003; 0.065)	0.182	2.132	**0.034** *
Cortisol concentration	−0.00008 (−0.0005; 0.0003)	−0.021	−0.358	0.721
CES-D sum score	0.018 (0.003; 0.034)	0.139	2.294	**0.022** *
GAD-7 sum score	0.032 (−0.007; 0.071)	0.099	1.626	0.105

b, Regression coefficient; *β*, Standardized regression coefficient; BMI, Body Mass Index; CES-D, Center for Epidemiological Studies Depression Scale (49, 50); CI, confidence interval; GAD-7, Generalized Anxiety Disorder 7-item Scale (51, 52); N/n, sample sizes; YFAS, Yale Food Addiction Scale (39). *p ≤ 0.05; **p ≤ 0.01; ***p ≤ 0.001. Significant findings were marked in bold.

YFAS subscale sum scores for desire, amount and continuous use were not significantly associated with total leptin concentrations in the total sample. The same was true for women. In the subgroup of men, there was a significant positive association between the YFAS subscale sum score for continuous use and the total leptin concentrations (*b* = 0.009; 95% CI: 0.001–0.017; standardized *β* = 0.141; *t* = 2.352; *p* = 0.019) whereas the associations between the total leptin concentrations and the YFAS subscale sum scores for desire and amount failed to be statistically significant.

All univariate multiple linear regression models have been adjusted for age, sex (only in the total sample), alcohol consumption, smoking status (1 = active smoking; 0 = non-smoking), BMI scores, cortisol concentrations, CES-D sum scores and GAD-7 sum scores.

These findings were in line with the results of extreme group comparisons (see Table 5): Men in the highest quartile of leptin serum levels were found to have significantly higher YFAS sum scores than men in the lowest quartile of leptin serum concentrations (1.55 vs. 1.18; *Z* = −3.81; *p* = 0.00014). For women the corresponding difference failed to be significant (*Z* = −1.53; *p* = 0.126).

**Table 5:** Extreme-group-comparison of food addiction intensity in the highest and lowest quartiles of leptin serum levels.

Quartiles	Leptin serum levels in pg./ml		Mean YFAS scores (SD) (range)		*N*	
	Men	Women	Men	Women	Men	Women
Lowest: <	2.705	8.20	1.18 (0.65) (0–4)	1.22 (0.62) (0–3)	132	95
Highest: >	7.99	24.19	1.55 (0.98) (0–7)	1.53 (1.03) (0–5)	133	97
---	---	---	*Z* = −3.806^a^ *p* = 0.0001 ***	*Z* = −1.529^a^ *p* = 0.126	---	---

SD, Standard Deviation; YFAS, Yale Food Addiction Scale (39). ^+^*p* < 0.10; *p < 0.05; **p < 0.01; ***p < 0.001. ^a^A Mann–Whitney U test was performed to assess group differences in YFAS scores due to non-normal distribution of the dependent variable (Kolmogorov–Smirnov-Test: *p* < 0.05).

## 4. Discussion

To our knowledge, this is the largest cross-sectional association study investigating the relationship between total serum ghrelin and leptin levels with FA-scores as assessed by the YFAS to date. We found that leptin serum levels were significantly associated with YFAS total score in men. However, it must be emphasized that this association was rather weak and occurred only in the exploratory analysis. In an extreme group comparison, male subjects with leptin levels in the highest quartile had significantly higher FA scores than subjects in the lowest quartile. No significant association was found in the total group and in women. In female subjects, the difference did not reach statistical significance. Total serum ghrelin did not show a significant association with FA-scores neither in the total group nor in men or women when assessed differentially.

There is ongoing debate on whether FA should be classified as a behavioral or substance addiction (6, 53, 54). While there is consensus that the consumption of food can have addictive properties (53), some authors see the origin of these addictive characteristics in behavioral processes, like in gambling (54), while others interpret the addictive properties of highly palatable foods as a key factor (6). This is based on the observation that subjects usually show addictive eating patterns with foods rich in sugar, processed carbohydrates and fats (6).

Our results are in line with the limited literature on this topic. For leptin, more data exists on its association with FA, with heterogeneous results. Altogether seven studies investigated this, with three studies finding significant relationships. A study in children and adolescents with the double burden of malnutrition (overweight but undersupplied with nutrients) found a positive relationship between leptin levels and FA scores in the overweight group of malnourished subjects (34). Likewise, in subjects suffering from MDD disordered eating was also positively associated with leptin serum levels (31) and leptin levels were higher in adolescent psychiatric inpatients with FA than in normal weight patients (35). In three other studies, no significant association between leptin and food addiction was found (5, 29, 32). In the study by Pedram et al., however, results were not controlled for BMI, age or sex, limiting the interpretability of the data. Furthermore, approximately 80% of subjects were female (29). Skinner et al. investigated only women, where we did not find a significant difference in leptin levels either (32). Tran et al. had a highly selective patient sample with only anorexia nervosa patients, of which 97% of patients were female (5). While leptin levels were not different in patients with and without FA, they were positively associated with emotional eating in patients with FA in another study (55). Thus, those studies that did not find a significant association between leptin and FA investigated mainly or only female subjects. Their results are in line with ours, as we did not find a significant association of leptin with FA in women either.

When looking at the association of leptin with BED, the majority of studies did not find any significant association (29, 56–60). Two studies described a significant positive relationship between leptin and uncontrolled eating (61, 62). The study by Bryant and colleagues was performed in obese patients who were candidates for bariatric surgery (61). 180 obese, pre-menopausal women were recruited by Yagin and colleagues (62). 41.6% of these were diagnosed with BED and higher leptin levels were a predictor for the presence of BED, after controlling for BMI (62). The cohort investigated in this study is highly selective and can only be compared to our population-based approach to a very limited degree (62). In conclusion, studies investigating leptin and FA and/or BED are very heterogeneous in their design, considerably limiting comparability. More high-quality studies with high standardization are needed to draw sound conclusions.

Few studies investigated the involvement of leptin in other behavioral addictions. On a whole, and in contrast to studies investigating ghrelin, no significant associations were found between leptin levels and gambling or decision-making (63, 64).

Interpreting our results with the back-drop of the literature up to date, one possible interpretation is that total serum ghrelin might not be a suitable biomarker to allow any conclusions about the absence or presence of food addiction. As already outlined above, most studies did not find a significant association between ghrelin and FA and BED. However, study heterogeneity was pronounced and similar, comparable designs to our study are lacking. Thus, while results thus far do not point to a major association of ghrelin with FA scores, more large-scale and high-quality studies are needed addressing this question.

For leptin, the positive association we found with FA scores in men could be interpreted as a sign for leptin resistance, where, despite high leptin levels, subjects still continue to consume food. Physiologically, leptin is involved in energy homeostasis by inhibiting hunger and food intake through central mechanisms and also by increasing metabolism (65). However, obese subjects continue to take in more food than needed despite higher than physiological leptin levels (65). This observation has led to the concept of central leptin resistance, the exact mechanism of which is still not entirely unraveled (65). It is, however, propagated to be a key pathogenic factor of obesity (65). In line this is our finding that the sub-scale of the YFAS which showed a positive association with leptin levels in men was ‘continuous use’, suggesting that despite high leptin levels, subjects had difficulties terminating food consumption. While more studies are needed corroborating this result in other cohorts, it would be an intriguing finding that this is an effect seen not just in obesity, but also in subjects with symptoms of food addiction, i.e., that is independent of the BMI and body fat content.

The sexual dimorphism, i.e., that leptin shows different effects in men and women, has been repeatedly described before. For the regulation of food intake, metabolism and energy homeostasis, it has been consistently shown that women show a higher central leptin sensitivity than men (66–69). The exact mechanism is unclear, but it is hypothesized that both inherent sex differences, mediated by X-chromosomal effects and neuroanatomical differences (70) as well as protective effects of 17*β*-estrogen play a key role in this phenomenon (71). Interestingly, this effect is not only present in appetite regulation and energy homeostasis but has been observed in MDD and/or depressive symptomatology as well (72–74).

In the four studies investigating the association of ghrelin with FA to date (29–32), only one study found a significant positive association (30). However, the heterogeneity of the studies published thus far considerably limits the conclusions that can be drawn from the results. Studies were heterogeneous as to whether total (31, 32) or acylated (29, 30) ghrelin was measured, what body weight ranges subjects had, and whether subjects were fasted or not as Mills et al. (31) investigated non-fasted individuals (31). Furthermore, two studies had only small sample sizes of 59 (29) and 19 (32). While our results add to the notion that FA scores might not be associated with total ghrelin levels, more large, high-quality studies are needed that measure acyl ghrelin. One possible interpretation of the current literature is that acyl ghrelin might be associated with higher FA scores, as demonstrated by Lopez-Aguilar and colleagues (30), and that the only other study investigating acyl ghrelin might have been underpowered (29). More evidence to this end exists when looking at studies investigating the involvement of ghrelin in BED, which shows a strong overlap with FA (4, 10). Here, most studies show no associations between BED and total ghrelin levels (75–78). However, two studies investigating acyl-ghrelin found significant associations, albeit negative ones (79, 80), with BED scores. Together, these results suggest that acyl-ghrelin might be significantly associated with both FA and BED-scores, while overwhelming evidence suggests that this is not the case for total ghrelin. More high-quality studies with large sample sizes measuring acyl-ghrelin are needed to further shed light on this subject.

As outlined above, FA is considered a behavioral addiction by some authors (4, 53). Ghrelin’s involvement in other behavioral addictions is poorly studied, with some evidence existing for patients suffering from gambling addiction. Ghrelin levels increased when subjects who gambled regularly were presented with gambling-related cues (81). Furthermore, acyl ghrelin levels were the strongest predictor for continued gambling despite continued loss. In a recent study, gamblers had higher levels of acyl ghrelin than healthy controls (82), and acyl ghrelin was positively associated with better performance in a sub-test of the Iowa Gambling Task (IGT) in healthy controls (82). This is in line with another recent study, showing a positive association between acyl ghrelin levels and performance in the IGT (63). Thus, the limited evidence that exists on ghrelin and behavioral addictions suggests that there might be an association with acyl ghrelin levels. Nevertheless, more high-quality studies are needed to further address this issue.

In our analyses, FA scores showed clear associations with age, BMI and depression scores (assessed by the CES-D) in both men and women. In females, FA scores were also associated with alcohol consumption and in the whole group FA scores were associated with anxiety as assessed by the GAD-7. For anxiety and depression, this is in line with pre-existing literature, showing increased rates of FA in depression (83, 84) and anxiety disorder (85). The fact that BMI is positively associated with YFAS scores is also well documented (86). However, for alcohol use, the limited data available thus far did not find significant associations between FA and alcohol consumption (86–89). The fact that we found a positive association between FA-scores and alcohol consumption in females might be an initial indication that when investigating the association of FA and alcohol consumption, a sex-stratified analysis should be considered.

The prevalence of manifest FA, as defined by the cut-off values of the YFAS, in our sample was very low at 1.1% (*n* = 10). Men and women were equally affected (*N* = 5 each). This is in contrast with the results from other epidemiological studies, which reported prevalences of FA of 9.4% (90), 15% (91) and 7.9% in a German sample (92). The exact reasons for this discrepancy remain speculative. However, one possible explanation might be the age profile of the cohorts. We investigated subjects aged between 40 and 79 with a mean age of 56.7 years. We could also show that YFAS scores and age were quite strongly negatively associated, meaning that scores were higher the younger the subject was. Nonetheless, the very low prevalence of FA in our sample has to be taken into consideration when interpreting our data.

The main strengths of this investigation were the large sample size and the highly standardized data acquisition. Furthermore, in contrast to other studies, we strictly controlled our statistical analysis for factors known to influence the ghrelin and leptin serum levels, most importantly in this context of anxiety scores (48), but also alcohol consumption (18) and smoking (42).

Limitations of this study are the fact that only total and not acylated ghrelin was measured and that blood samples were not pre-treated with protease inhibitors or acidification. The cross-sectional design does not allow for causal conclusion and FA scores were only assessed by a self-assessed screening instrument. In self-assessment instruments, response behavior should be taken into consideration. A tendency toward socially desirable answers might decrease YFAS scores, while worrying about one’s eating behavior and weight might produce higher YFAS scores, even with no FA present. Furthermore, the prevalence of FA was markedly lower than in comparable cohorts, which should be considered when interpreting the data.

In conclusion, our large-scale, population-based approach to investigating the association between FA scores and ghrelin and leptin adds new insight to existing literature. We provided further evidence to the observation that FA scores are not strongly associated with ghrelin serum levels. For leptin, we could show a positive association with FA scores for men in a large, community-based approach for the first time.

## Data availability statement

The raw data supporting the conclusions of this article will be made available by the authors, without undue reservation.

## Ethics statement

The studies involving human participants were reviewed and approved by Ethics committee of the University of Leipzig (registration-number: 263-2009-14122009). The patients/participants provided their written informed consent to participate in this study.

## Author contributions

DW: conceptualization and design, validation, project administration, and writing—original draft. JK: investigation and writing—review and editing. RM: validation, statistical analysis, and writing—review and editing. KW: investigation, resources, funding acquisition, and writing—review and editing. AW: funding acquisition, resources, and writing—review and editing. AV: funding acquisition and project administration. MK: conceptualization and design, validation, project administration, writing—original draft, and supervision. All authors contributed to the article and approved the submitted version.

## Funding

This work was supported by LIFE − Leipzig Research Center for Civilization Diseases, University of Leipzig. LIFE is funded by means of the European Union, by means of the European Social Fund (ESF), by the European Regional Development Fund (ERDF), and by means of the Free State of Saxony within the framework of the excellence initiative. Part of the work was funded within the framework of the Collaborative Research Center “Adiposity Mechanisms” 209933838/Deutsche Forschungsgemeinschaft (DFG, German Research Foundation). The author(s) acknowledge support from the German Research Foundation (DFG) and Universität Leipzig within the program of Open Access Publishing.

## Conflict of interest

The authors declare that the research was conducted in the absence of any commercial or financial relationships that could be construed as a potential conflict of interest.

## Publisher’s note

All claims expressed in this article are solely those of the authors and do not necessarily represent those of their affiliated organizations, or those of the publisher, the editors and the reviewers. Any product that may be evaluated in this article, or claim that may be made by its manufacturer, is not guaranteed or endorsed by the publisher.
